# Categorisation of culturable bioaerosols in a fruit juice manufacturing facility

**DOI:** 10.1371/journal.pone.0242969

**Published:** 2021-04-21

**Authors:** Shirleen M. Theisinger, Olga de Smidt, Jan F. R. Lues

**Affiliations:** Centre for Applied Food Sustainability and Biotechnology (CAFSaB), Faculty of Health and Environmental Sciences, Central University of Technology, Free State, Bloemfontein, South Africa; COMSATS University Islamabad - Abbottabad Campus, PAKISTAN

## Abstract

Bioaerosols are defined as aerosols that comprise particles of biological origin or activity that may affect living organisms through infectivity, allergenicity, toxicity, or through pharmacological or other processes. Interest in bioaerosol exposure has increased over the last few decades. Exposure to bioaerosols may cause three major problems in the food industry, namely: (i) contamination of food (spoilage); (ii) allergic reactions in individual consumers; or (iii) infection by means of pathogenic microorganisms present in the aerosol. The aim of this study was to characterise the culturable fraction of bioaerosols in the production environment of a fruit juice manufacturing facility and categorise isolates as harmful, innocuous or potentially beneficial to the industry, personnel and environment. Active sampling was used to collect representative samples of five areas in the facility during peak and off-peak seasons. Areas included the entrance, preparation and mixing area, between production lines, bottle dispersion and filling stations. Microbes were isolated and identified using 16S, 26S or ITS amplicon sequencing. High microbial counts and species diversity were detected in the facility. 239 bacteria, 41 yeasts and 43 moulds were isolated from the air in the production environment. Isolates were categorised into three main groups, namely 27 innocuous, 26 useful and 39 harmful bioaerosols. Harmful bioaerosols belonging to the genera Staphylococcus, Pseudomonas, Penicillium and Candida were present. Although innocuous and useful bioaerosols do not negatively influence human health their presence act as an indicator that an ideal environment exists for possible harmful bioaerosols to emerge.

## Introduction

Bioaerosols are defined as “aerosols comprising of particles of biological origin or activity which may affect living things through infectivity, allergenicity, toxicity and pharmacological or other processes [1–3]. Bioaerosols are emerging as important role players in atmospheric processes, yet they are poorly understood. What is known and is universally accepted is that bioaerosols originate from and may impact various environments. For example, bioaerosols are emitted from terrestrial sources such as soil, forests and desert dust, and from agricultural and composting activities. They are prevalent in urban areas, wetlands, as well as coastal and marine environments. Moreover, they play a key role in the dispersal of reproductive units from plants and microbes where the atmosphere enables their dissemination over geographic barriers and long distances. Bioaerosols are thus highly relevant in the spread of organisms and they allow genetic exchange between habitats and geographic shifts of biomes. These compounds are central elements in the development, evolution and dynamics of ecosystems [4, 5]. Although bioaerosols may have beneficial characteristics, the opposite is also possible, as the dispersal of plant, animal and human pathogens as well as allergens has major implications for agricultural outcomes and public health. The negative effects that bioaerosols may have on the human respiratory system are documented [6, 7]. With research that has focused on fungal pathogens and bacterial bioaerosols represent an urgent research priority due to their role in disease outbreaks [8].

Modern industrial activities (e.g., waste sorting, organic waste collection, composting, agricultural production, food processing, raising of livestock, and wastewater treatment systems) emit large quantities of bioaerosols, and this results in the release of abundant biological agents into the air. Unfortunately, it is difficult to accurately describe bioaerosols role on the environment, especially in terms of human health [5, 9], and thus the effects that bioaerosols may have on products and food handlers in the food industry remain controversial. To exacerbate this situation, no legislation is available regarding bioaerosols in the air of food industries in South Africa. Allowable quantities of bioaerosols as proposed by the European Union have been disseminated, but there is no set standard [10]. What makes the assembly of legislation for bioaerosols so difficult is the fact that, in a specific industry, two or more manufacturing facilities might produce the same product, but the environment, other industries in close proximity, the season, the structure of the facility, and the raw materials used can differ to such an extent that the bioaerosol composition may vary considerably among these facilities [11].

In a generic food facility, major routes of food contamination by microorganisms are via surface contact, via personnel or via the air. The contamination by air is noteworthy for products such as beverages, refrigerated dairy and culinary products [12]. Monitoring bioaerosols in the food industry environment is one of the many tools that industrial quality control managers can use in the assessment of indoor air quality, agricultural outcomes, and industrial health. The monitoring process should include: (i) sampling of bioaerosols using either passive or active sampling methods; (ii) measurement of viable (culturable and non-culturable) and non-viable bioaerosols; and (iii) the identification of bioaerosols. Identification of microbial taxa is a critical element in the determination of the bioaerosol load in an industrial environment. Identification of bioaerosols can be performed using a variety of available assessment strategies such as microscopy, immuno-assays as well as various molecular-based assays [13–15]. The sensitivity and rapidity of molecular techniques have also led to their use for bioaerosol monitoring in the determination of air quality and the detection of airborne pathogens [9, 16].

The air in food industries can be packed with various airborne microorganisms that may include bacteria, yeast and mould [9, 17].The contribution of airborne microorganisms to food contamination has been addressed, although aerosols in food plants have not been studied sufficiently too accurately generalise particle distribution [18]. The compilation of organisms in the air depends on the industry, the facility, the capacity of the facility, as well as the season and the external environment. Airborne microorganisms are a potential source of a wide variety of public and industrial health hazards; however, it is difficult to compile a set standard of acceptable limits for a specific industry as information regarding; due to difference in samplers, collection time, airflow rate, analysis method and the types of bioaerosols and their effects is not abundant [8, 11]. With limited information the aim of this study was to characterise the culturable fraction of bioaerosols sampled during peak and off-peak seasons in a fruit juice manufacturing facility and categorise isolates as harmful, innocuous or potentially beneficial to the industry, the personnel and the environment.

## Materials and methods

### Sampling

**Fig 1:**
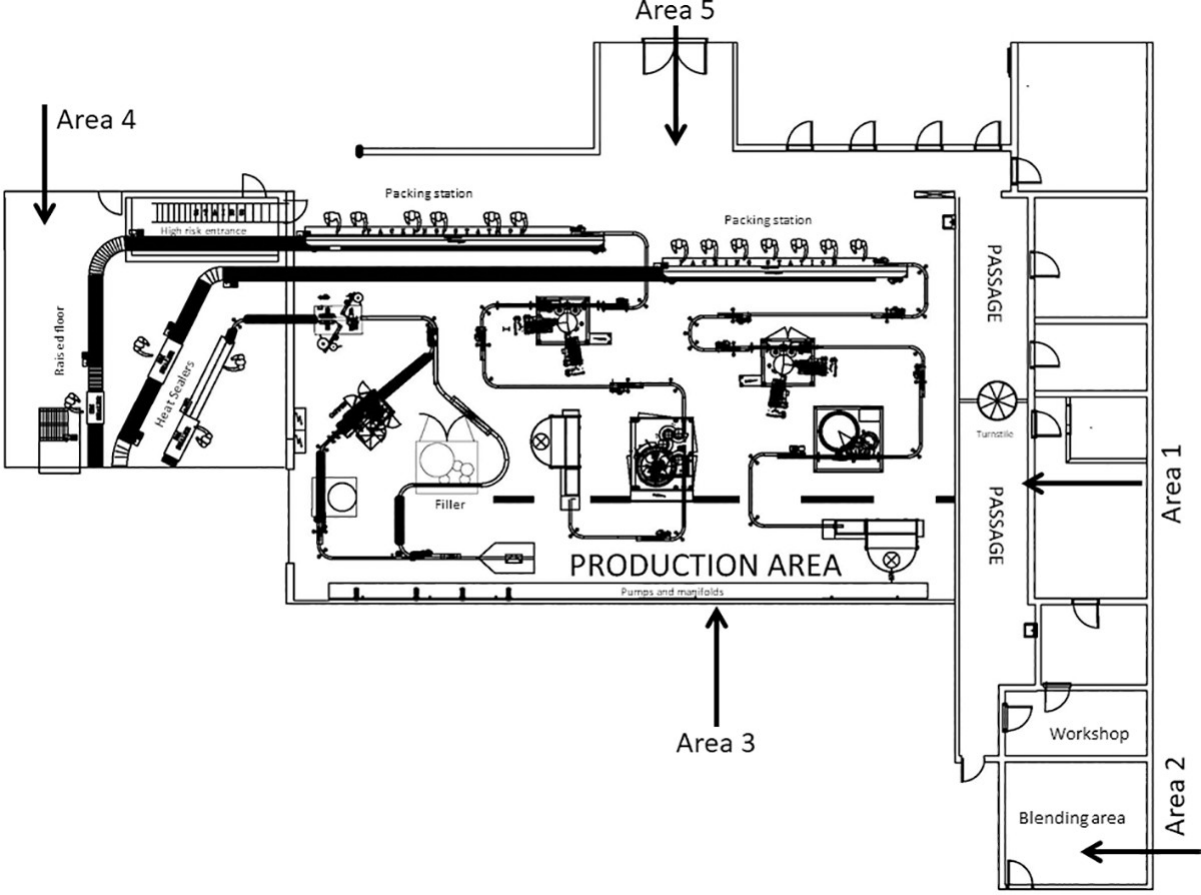
Schematic layout of the fruit juice manufacturing facility. Different sampling areas: Area 1 (entrance to the production area), Area 2 (preparation and mixing of materials), Area 3 (between the production lines), Area 4 (dispersion of bottles) and Area 5 (filling of bottles with the final product).

Two SAMPL’AIR LITE (AES Chemunex, United States) samplers were used to collect culturable bioaerosols in a HACCP certified fruit juice manufacturing facility in Bloemfontein, South Africa. A purposive sampling methodology was utilised [18] that was appropriate for the selected peak and off-peak manufacturing seasons according to which the facility operated. All sampling was performed in duplicate in the entrance to the production area (Area 1), the preparation and mixing area of materials (Area 2), the area between the production lines (Area 3), the area for the dispersion of bottles (Area 4), and the area where the bottles were filled with the final product (Area 5) (Fig 1).

The air samplers that were used operated at a flow rate of 100 L per minute and were disinfected with ethanol between the different sampling points. The samplers were switched on for 2 min prior to sampling to allow the ethanol to evaporate, thereby avoiding interference with the quantities of microorganisms recovered. Air samples were taken at a height of 1.5 m from the ground [19, 20], which was the same level as the working stations in the centre of each area. Sterile petri dishes containing either non-selective or selective media were used (Table 1). After a sampling time of 5 min, the samplers were switched off and the petri dishes were removed and inverted in their covers. True temperature was determined using a heat stress monitor IQuestemp SA) and a rotating vane anemometer was used for the measurement of air velocity and volume flow. At least two independent repeats were conducted in each sampling area.

**Table 1:** Media, incubation time and temperature.

Enumeration Conditions for Different Microorganisms			
Microorganisms	Media	Incubation Time	Temperature
Total microbial load	PCA (plate count agar)	72 h	30°C
Yeast and mould	RBC (Rose Bengal Chloramphenicol agar)	72–120 h	25°C
Coliforms and *E*. *coli*	VRB (Violet Red Bile agar) with MUG (4-Methylumbelliferyl-β-D-Glucuronide)	24 h	37°C
*Salmonella* spp.	XLD (Xylose Lysine Deoxycholate agar)	24 h	37°C
*Staphylococcus* spp.	BPA (Baird-Parker agar)	48 h	37°C

### Culture medium composition used for microbe cultivation, enumeration and isolation

Active air samples collected on the petri dishes containing the non-selective and selective media were incubated for a specific time at specific temperatures [5] (Table 1).

Selective media are predominantly used for the growth of selected microorganisms. Microbial counts were performed using standard guidelines adapted from *The Compendium of Methods for the Microbiological Examination of Foods* [21, 22]. After incubation, the number of colonies on each plate was counted using the Scan® 1200 high-resolution automatic colony counter. The colony counts were adjusted using the positive hole correction method based on Feller’s statistical correlation equation [23, 24] and reported as CFU.m^-3^. Individual presumptive bacterial colonies were selected with a sterile inoculation loop and preserved in 2 ml Microbanks (ProLab) at -80°C. Yeast and mould colonies were stored in 1.8 ml Nunc^®^ CryoTubes^®^ containing 1 ml sterile 15% glycerol at -80°C.

### Identification of the culturable fraction of bacteria, yeast and mould

Pure cultures of bacteria, yeast and mould were selected from 18 to 72 h agar plates based on colony colour, morphology and cell characteristics using a microscope [25, 26]. Selected colonies were purified onto fresh agar plates and whole cells used for PCR identification. Primer set 63F (5’-CAG GCC TAA CAC ATG CAA GTC-3’) and 1387R (5’-GGG CGG WGT GTA CAA GGC-3’) were used to target ≈1 300 bp of the 16S rRNA gene for bacterial identification [27]. Primers NL1 (5’-GCA TAT CAA TAA GCG GAG GAA AAG-3’) and NL4 (5’-GGT CCG TGT TTC AAG ACG G-3’) were used for the amplification of the D1/D2 domain of the 26S rRNA gene (≈600 bp) of yeasts [28, 29]. For mould identification, a PCR-mediated reaction was performed targeting the ≈600 bp internal transcribed spacer region (ITS1, ITS2) using primers ITS1 (5’-TCC GTA GAA CCT GCG g-3’) and ITS4 (5’-TCC TCC GCT TAT TGA TAT GC-3’) (Integrated DNA Technologies, Inc.) [30–32].

Whole cells from a pure culture suspension (20 μl) were used as template DNA. The PCR was carried out in a total volume of 50 μl, containing 1X ThermoPol® reaction buffer (20 mM Tris-HCI, 10 mM (NH_4_)_2_SO_4_, 10 mM KCI, 2 mM MgSO_4_, 0.1% Triton®-X-100, pH 8.8 @ 25°C), 0.2 mM dNTPs, 0.52 μM of each primer and 1 unit of Taq DNA polymerase (New England Biolabs). PCR reaction conditions included an initial denaturation cycle of 3 min at 95°C, followed by 30 cycles of denaturation at 95°C, annealing at 55°C for 30 sec and elongation at 68°C for 60–90 sec. A final elongation cycle was performed at 68°C for 6 min. PCR products were separated on a 1% agarose gel, stained with 0.05% Ethidium bromide, and visualised using UV light. Digital images were captured with the Molecular Imager® Gel Doc™ XR system (BioRad Laboratories, Inc.).

After purification using the Diffinity RapidTip®2 (Sigma), both forward and reverse primers were used for sequencing in separate reactions [4]. Sequencing was performed using the ABI Prism 3130 XL genetic analyser and the Big Dye® Terminator V3.1 Cycle Sequencing Kit (Applied Biosystems). DNA was precipitated with EDTA and ethanol. Contigs of forward and reverse sequence results were assembled using DNA Baser v5.15.0 sequence assembly software and compared with sequences accessible in the GenBank database using the BLAST algorithm (megablast) [4, 33]. Sequences with high similarity were then subjected to multiple sequence alignments using Clustal Omega (EMBL-EBI) for identification [34]. Only similarities with a BLAST index of 97% and above were considered for identification [35]. Sequence data for isolates considered harmful were deposited into the National Center for Biotechnology Information (NCBI) Genbank database. Genbank accession numbers were included in Table 4.

## Results and discussion

### Culturable fraction identified during peak and off-peak sampling

Airborne microorganisms occur ubiquitously in ambient air [36] and are naturally part of the air in almost any environment. These microbes can originate, not only from humans, but are also spawned by various indoor characteristics (such as ventilation, heating and air conditioning systems) and outdoor environmental sources. Although airborne microorganisms encountered in indoor facilities are still deemed innocuous for healthy individuals, they can cause adverse health effects when high counts are ingested or inhaled [37, 38]. Moreover, bioaerosols are easily translocated from one ecosystem to another by wind and air currents, thus making them an important vehicle for the spread of potentially pathogenic organisms [39]. When associated with dust particles or condensation droplets, these organisms can be dispersed among different areas in a food processing unit. International food industries are required by authorities such as the Food and Drug Administration (FDA) to take measures to reduce product contamination by airborne microorganisms [40, 41].

**Fig 2:**
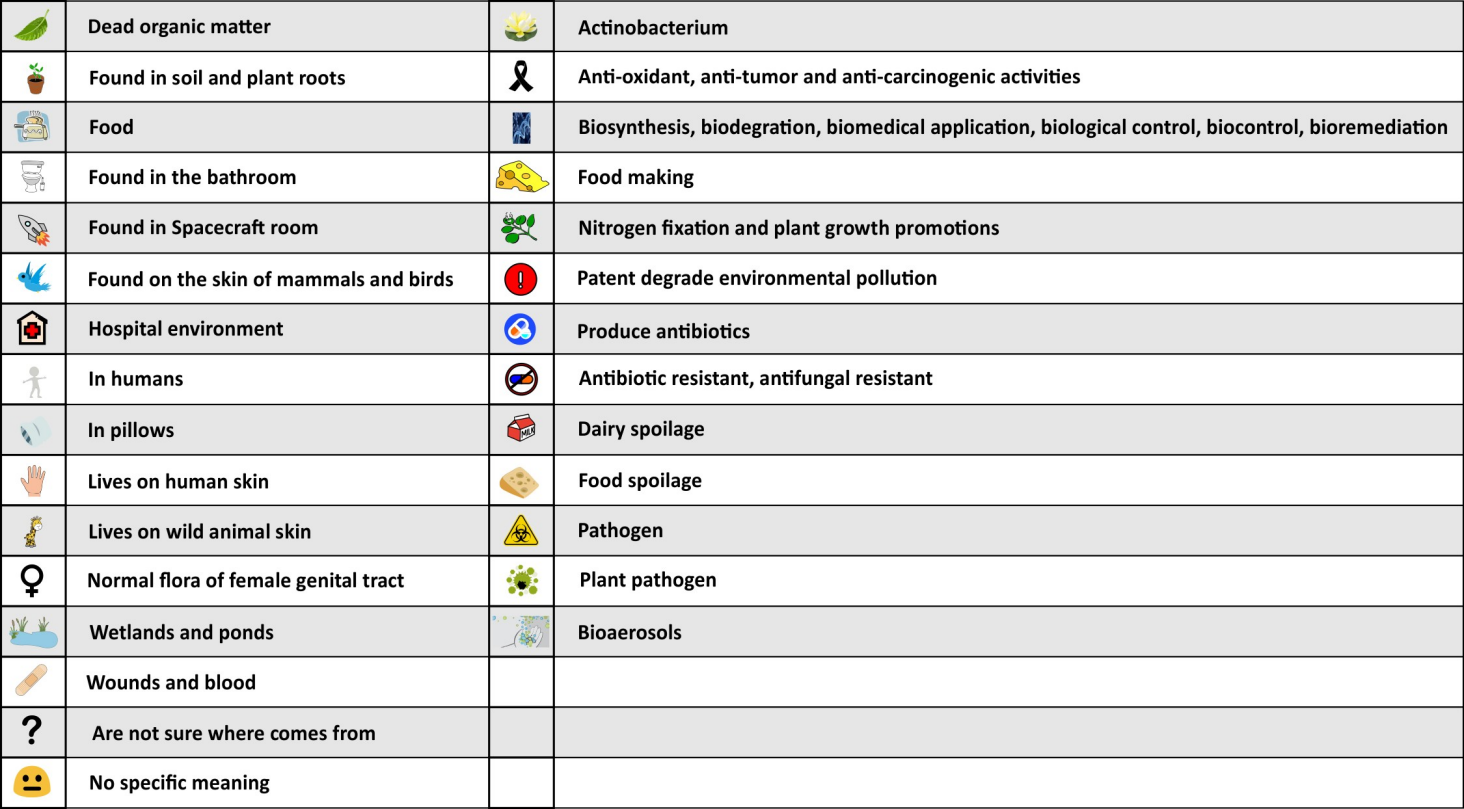
Symbol key. These symbols are used in Fig 3 to link the microorganisms to their possible origins (from ‘Dead organic matter’ to ‘Wounds and blood), interest (from “Not sure where it comes from’ to ‘No specific meaning’), and importance (from ‘Actinobacterium’ to ‘Bioaerosols’).

**Fig 3:**
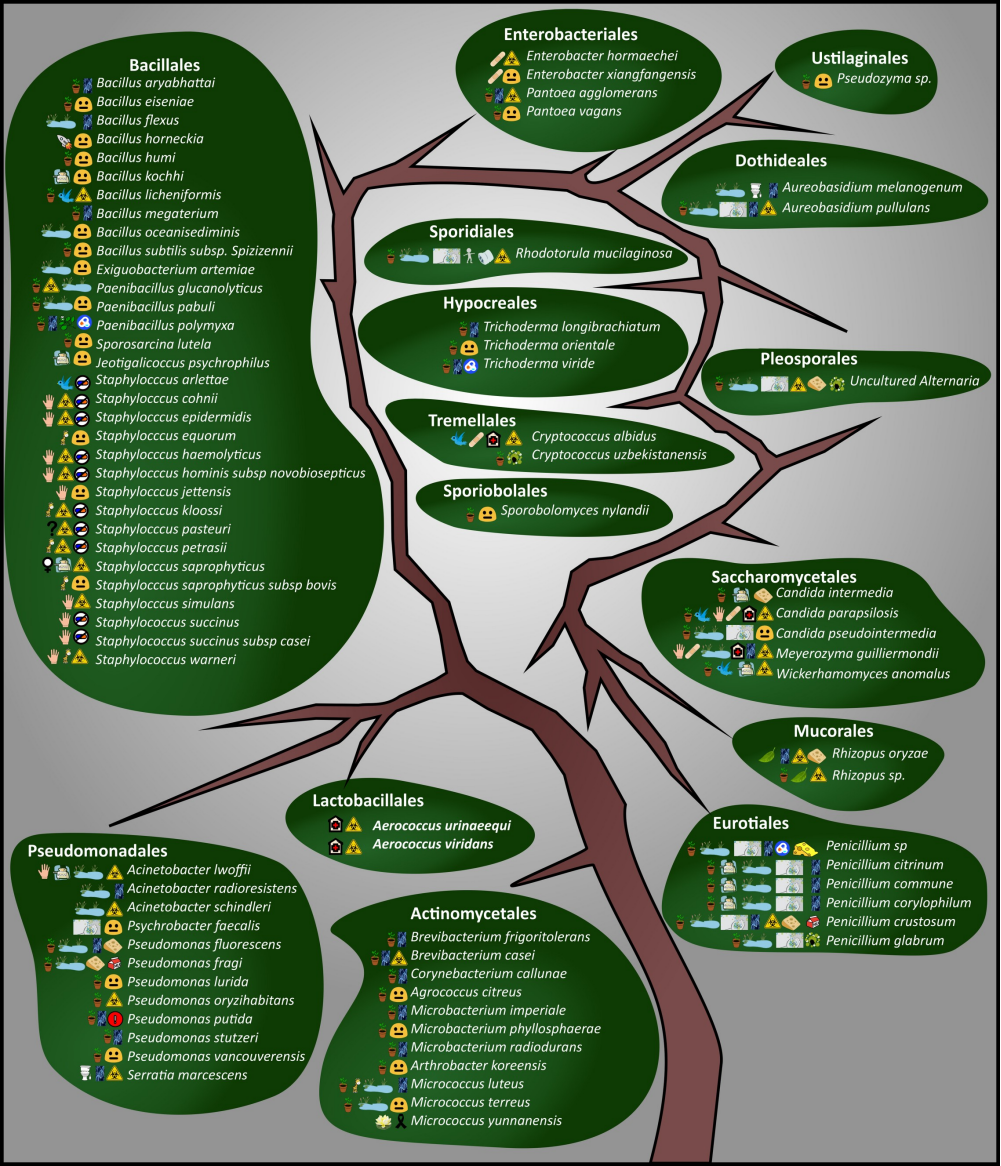
Bioaerosol distribution tree. Overview of the culturable bioaerosol fraction. As this is a distribution tree, each order is shown as different leaves and the various taxa are indicated in italics. The meaning of each symbol is outlined in Fig 2. This is not a phylogenetic tree, nor is there a specific listing order; it merely represents the total diversity detected. Microbial orders are discussed from the bottom left starting with the Actinomycetales in a clockwise direction under the two different kingdoms. The Bacillales, Pseudomonadales and Actinomycetales, and to a lesser extent the Eurotiales and Saccharomycetales orders, were the most prevalent.

Bacteria, yeast and mould are the main groups of microorganisms categorised as potential pathogenic airborne microorganisms. Bacteria, yeast and mould have been identified in various food industries as bioaerosols. These industries include dairy processing facilities [42], poultry slaughtering facilities [43], automated chicken egg production facilities [44], and bakeries [45]. During our study we isolated a total of 239 bacteria and 41 yeasts and 43 moulds from the air in the production environment of the fruit juice manufacturing facility. An overview of these bioaerosols is presented as a distribution tree where the bacteria, yeast and mould are classified into different phylogenetic orders (Figs 2 and 3). From the isolates obtained, 86 different species belonging to 15 different taxonomic orders representing five bacteria and ten yeast and mould orders were identified.

Bacteria are the most abundant and diverse group of organisms [46] and are ubiquitous in every habitat on Earth. They can be present in soil, water and organic matter as well as in live bodies of plants and animals. A number of bacterial species presence in indoor environments is mostly related to human occupancy and type of indoor environment [47]. Moreover, bacteria are abundant in the atmosphere where they often represent a major portion of organic aerosols [48]. Even though bacteria were represented by a smaller group of orders in this study, they represented 62 different species.

It was immediately visible in Fig 3 that many bioaerosols detected in the facility probably originated from soil and plant roots, wetlands and ponds, and human skin. Many species that were detected had no specific significance; however, a reasonable quantity could be considered as pathogenic, specifically in the order Bacillales that is antibiotic resistant. Mainly fungal isolates and one *Pseudomonas* have previously been described as bioaerosols. Only four fungal and two bacterial isolates have previously been associated with food poisoning/spoilage.

All species found in the order Actinomycetales are found in soil and plant roots. Of these species, *Micrococcus* seems to be a predominant species in indoor air [47, 49]. The order Pseudomonadales was represented by the genera *Acinetobacter* spp., *Psychrobacter* spp. and *Pseudomonas* spp., most of which are found in soil, plant roots, wetlands and ponds. Evidence indicates that bioaerosol agents such as *Pseudomonas* spp. cause occupational health threats to immuno-compromised patients [50]. *Psychrobacter faecalis* is one species of this order that was discovered in a bioaerosol originating from pigeon faeces [51].

The order Lactobacillales represents a morphologically, metabolically and physiologically diverse group of bacteria [52]. In the current study only one genus, *Aerococcus*, with two different species was identified. Both these species are classified as pathogenic. They are prevalent in hospital environments and can form biofilms [53]. The order Bacillales represents a wide variety of different species with a wide variety of origins and interests. Genera from the order Bacillales are frequently found to be part of bioaerosols, because genera such as *Bacillus* and *Paenibacillus* can form highly tolerant endospores that can travel long distances [47, 54]. Two main genera were identified, namely *Bacillus* and *Staphylococcus*, both known to form part of bioaerosols. They also possess pathogenic abilities and can be resistant to antibiotics [47, 50, 55, 56]. From the Enterobacteriales order, three different genera were identified, namely *Enterobacter* spp. and *Pantoea* spp. (both are found in soil and/or plant roots and in wounds and blood) as well as *Serratia* spp. (which is mostly found in bathrooms). *Pantoea agglomerans* is usually associated with plants and is seen as a bacterium of good and evil, implying it can contribute to plant growth or cause opportunistic infections in humans [57].

Eukaryotic cells are considerably more complicated than those of prokaryotic origin and are characterised by a high degree of cellular complexity (lysosomes, peroxisomes, microtubules, mitochondria, cytoskeleton, etc.), which makes the classification of these microorganisms quite difficult [58]. This may explain why yeast and mould could be classified in 10 different orders whereas only 23 different species were identified. Overgrowth of mould in the petri dishes was observed during the enumeration of the culturable fraction and lower yeast and mould counts were observed compared to total microbial load. This may also have contributed to a lower number of identified species, as the overgrowth may have prevented accurate isolation of other moulds and yeasts that could have been present [59, 60]. *Pseudozyma*, a yeast found mainly in soil and plant roots [61], was the only genus detected from the order Ustilaginales. Of the order Dothideales (microorganisms found mostly in soil, plant roots, wetlands and ponds), only one of the species, *Aureobasidium pullulans*, was previously classified as a bioaerosol [62].

Pleosporales is the largest order in the Dothideomycetes species and it comprises a quarter of all the detected Dothideomycetous species. Species in this order occur in various habitats, including bioaerosols [63]. From the Pleosporales order, one genus was detected, namely an uncultured *Alternaria* spp. that can originate from a large variety of environments such as soil plant roots, wetlands and ponds. The *Alternaria* spp. is seen as a plant pathogen; however, reports have stated that it is also prevalent in the food industry [64]. Three well known genera from the order Saccharomycetales were detected: (i) *Candida* spp. (one specific *Candida* species, *Candida pseudointermedia*, was previously identified as a bioaerosol), that originates from soil and plant roots and has the ability to cause invasive fungal infection that can have a significant impact on public health [65, 66]; (ii) *Meyerozyma guilliermondii*, which is known for its ability to live on human skin and in wounds and blood and has spoilage abilities [67]; and (iii) *Wickerhamomyces anomalus*, which is found in food and has pathogenic abilities [68]. The order Mucorales was represented by only one genus, namely a *Rhizopus* spp. This genus is mostly found in dead organic matter and has pathogenic abilities [69].

Eurotiales are widespread and abundant fungi that include the well-known genus *Penicillium*. *Penicillium* is recognised as one of the most abundant mould genera in indoor air [47, 65, 70]. Similarly, in the order Sporobolales, only one species was detected, namely *Sporobolomyces nylandii*, which is normally found in soil and/or plant roots [71]. *Cryptococcus* spp., from the order Tremellales, were also identified. These species are known to have either human or plant pathogenic abilities and have been identified as bioaerosols [65, 72]. From the Hypocreales order three different *Trichoderma* spp. were detected, and all originate from soil and/or plant roots [73–75]. From the Sporidiales order only *Rhodotorula mucilaginosa was identified*. This organism is found in soil, plant roots, wetlands, ponds, and in humans and on/in pillows. *Rhodotorula mucilaginosa* has been receiving increased attention because it can be isolated from extreme ecosystems and has the capability to survive and grow in many unfavourable conditions. It is also classified as a bioaerosol and a human pathogen [76].

Various microorganisms that were detected support the existing scientific literature that indoor exposure to microorganisms poses a risk for asthma and allergies among occupants of indoor facilities [77]. It is undeniable that microbial contamination of a facility has the potential to affect the product and places the occupants at risk of developing airway difficulties. Surprisingly, little research is available with regards to these microorganisms in the food industry, especially in the fruit juice industry, and therefore it was important to clearly classify the microorganisms that were identified. This will aid in better understanding the prevalence and ecology of specific indoor airborne bioaerosols. Furthermore, it will be a useful tool in the management and prevention of both long- and short-term problems faced in the fruit juice industrial setting [47].

### Classification of the bioaerosols detected

Bioaerosols are generated via multiple sources such as different instruments, external environments, and human activity. Bioaerosols have varying microbiological profiles depending on their origin and reason of interest and contaminate a product produced in an industry or pose a hazard to workers [50], depending on the kind of bioaerosol that is involved. Literature classifies bioaerosols into three groups, namely: (i) innocuous [78]; (ii) useful [4]; and (iii) harmful [79] After the identification of the culturable fraction of bioaerosols in the current study, it was concluded that 27 innocuous, 26 useful and 39 potentially harmful bioaerosols were detected. Several of these bioaerosols can be classified in more than one group depending on the environment and intended use of the facility/area.

#### Innocuous bioaerosols

Innocuous microorganisms were classified in 1985 by the European Federation of Biotechnology as “microorganisms that have never been identified as causative agents of disease in man and that offer no threat to the environment” [80]. For a microorganism to be described as environmentally safe, it should meet the following criteria: (i) be non-pathogenic to humans, animals and plants; (ii) must have a limited ability to compete; (iii) will not indirectly affect other species (by the production of toxic metabolites or biogeochemical changes); (iv) is unable to irreversibly alter equilibria between nutrients, microflora, and higher organisms; (v) is unable, in the open environment, to transfer genetic traits that can be noxious in other species; (vi) unable to cause food spoilage; and (vii) does not contribute to unwanted traits [4, 81].

We identified 27 different microorganisms as innocuous bioaerosols (Table 2). Two genera were dominant, namely *Bacillus* and *Staphylococcus*. The genus *Bacillus* includes more than 200 species, is widespread in nature and is found in virtually every environment [82]. Although the *Bacillus* species are ostensibly well-known as pathogens, the overwhelming majority are in actual fact non-pathogenic [83]. The *Staphylococcus* species are reported as normal microbiota of mammals and birds; however, certain species are important pathogens in humans and animals. It is noteworthy that little is known about the *Staphylococcus* species that are non-pathogenic environmental microorganisms [84].

**Table 2:** Innocuous bioaerosols detected and classified alphabetically from order to species.

Innocuous Bioaerosols–Bacteria			
Order	Family	Genus and Specie	Reference
Actinomycetales	Brevibacteriaceae	*Agrococcus citreus*	[89]
Actinomycetales	Microbacteriaceae	*Microbacterium phylloshaerae*	[90]
Actinomycetales	Micrococcaceae	*Arthrobacter koreensis*	[91]
Actinomycetales	Micrococcaceae	*Micrococcus terreus*	[92]
Bacillales	Bacillaceae	*Bacillus eiseniae*	[82]
Bacillales	Bacillaceae	*Bacillus horneckiae*	[93]
Bacillales	Bacillaceae	*Bacillus humi*	[94]
Bacillales	Bacillaceae	*Bacillus kochii*	[95]
Bacillales	Bacillaceae	*Bacillus oceanisediminis*	[92]
Bacillales	Bacillaceae	*Bacillus subtilis subsp*. *spizizenii*	[83]
Bacillales	Bacillaceae	*Exiguobacterium artemia*	[96]
Bacillales	Paenibacillaceae	*Paenibacillus pabuli*	[97]
Bacillales	Planococcaceae	*Sporosarcina luteola*	[98]
Bacillales	Staphylococcaceae	*Jeotgalicoccus psychrophilus*	[99]
Bacillales	Staphylococcaceae	*Staphylococcus equorum*	[100]
Bacillales	Staphylococcaceae	*Staphylococcus jettensis*	[101]
Bacillales	Staphylococcaceae	*Staphylococcus saprophyticus subsp*. *bovis*	[102]
Enterobacteriales	Enterobacteriaceae	*Enterobacter xiangfangensis*	[103]
Enterobacteriales	Erwiniaceae	*Pantoea vagans*	[104]
Pseudomonadales	Moraxellaceae	*Psychrobacter faecalis*	[51]
Pseudomonadales	Pseudomonadaceae	*Pseudomonas lurida*	[105]
Pseudomonadales	Pseudomonadaceae	*Pseudomonas vancouverensis*	[106]
Sphingomonadales	Sphingomonadaceae	*Sphingomonas pseudosanguinis*	[107]
Saccharomycetales	Saccharomycetaceae	*Candida pseudointermedia*	[108]
Hypocreales	Hypocreaceae	*Trichoderma orientale*	[74]
Sporiobolales	Incertae sedis	*Sporobolomyces nylandii*	[71]
Ustilaginales	Ustilaginaceae	*Pseudozyma spp*.	[61]

Only 4 innocuous yeasts and moulds were detected. Although yeast and mould are well-known for their fermentation ability and pharmaceutical properties, it has been found that they are microorganisms that do more harm than good in food and food-related industries [85–88].

Although these microorganisms would have been innocuous to the products, the workers in the facility and the environment, they still formed part of the bioaerosols detected during bioaerosol sampling. The high microbial counts that were observed during sampling immediately created the inaccurate assumption that the air was contaminated with hazardous or unsafe bioaerosols [109]. Therefore, simply analysing bioaerosols for total heterotrophic counts, as specified by certain countries to determine air quality, could be considered a shortcoming [110].

#### Useful bioaerosols

Useful microorganisms are generally: (i) environmentally beneficial; (ii) useful in food; (iii) making positive medical contributions; and (iv) biotechnologically advantageous. For example, the use of beneficial/useful microorganisms contributes positively towards environmentally safe agricultural products. The modes of action of these useful microorganisms and their various benefits to plants range from the simple occupation of biological empty spaces to ecological relationships such as antibiosis, competition, predation, and symbiosis, among others [111]. Other beneficial microorganisms represent an important biotechnological approach to decrease the deleterious effects of stress in crops [112, 113]. Studies have also indicated that the growth-promoting ability of some bacteria to synthesise extracellular polysaccharides or exopolysaccharides has commercially significant applications [114].

The use of beneficial microorganisms can potentially revolutionise agriculture and food industries by: (i) integrating crop health with better management practices for specific climatic conditions to improve productivity and quality; (ii) using environmentally friendly approaches to control pests and pathogens, thus reducing the use of chemical pesticides with environmental and health implications; (iii) producing better quality food with less chemical contamination and allergens; and (iv) minimising losses by improving crop fitness in extreme weather conditions [115].

One of the most exciting scientific advances in recent years has been the realisation that commensal microorganisms play key roles in our physiology (including protection against infection) and in drug metabolism, vitamin synthesis, nutrition, as well as in response to disease [116]. The beneficial influence of microorganisms is still on the border of its’ potential and a great deal of future discoveries and technologies are anticipated. In the current study, the useful bioaerosols that were detected during the selected sampling seasons were categorised into three groups, namely: (i) medical contribution; (ii) promoting and protecting plant growth; and (iii) environmental contribution (Table 3).

**Table 3:** Alphabetical classification of useful bioaerosols detected (peak and off-peak seasons) according to: medical contribution, promoting and protecting plant growth and environmental contribution.

Genus and Specie	Benefit	Reference
**Medical Contribution**		
*Acinetobacter radioresistens*	Purification and biochemical properties	[117]
*Bacillus flexus*	Capable of synthesis of anisotropic silver nanoparticles	[118]
*Bacillus megaterium*	Capable of biosynthesis of silver nanoparticles and have antibacterial activity on multi drug resistant clinical pathogens	[119]
*Brevibacterium frigoritolerans*	Capable of producing silver nanoparticles	[120]
*Corynebacterium callunae*	Have the function for activity and stability of the enzyme Orthophosphate	[121]
*Microbacterium radiodurans*	UV radiation-tolerant bacterium useful in cancer research, with heavy metal bioremediation capabilities	[122]
*Micrococcus yunnanensis*	Anti-oxidative, anti-tumour-promoting, and anti-carcinogenic activities of adonirubin and adonixanthin	[123]
*Meyerozyma guilliermondii*	Antifungal activity	[124]
*Penicillium corylophilum*	Antibacterial activity	[125]
Penicillium spp.	Capable of biosynthesis of silver nanoparticles	[126]
**Promoting and Protecting Plant Growth**		
*Bacillus aryabhattai*	Zinc-solubilising abilities	[127]
*Brevibacterium casei*	Capable of promoting plant growth	[128]
*Microbacterium imperial*	Capable of biodegradation of bromoxynil–to reduce its acute toxicity	[129]
*Paenibacillus polymyxa*	Capable of nitrogen fixation, plant growth promoting, soil phosphorus solubilisation and production of exopolysaccharides, hydrolytic enzymes, antibiotics and cytokinin. Helps bioflocculation and the enhancement of soil porosity as well as capable of producing optically active 2,3-butanediol (BDL)	[130]
*Pantoea agglomerans*	Capable of controlling post-harvest diseases on apples	[131]
*Pseudomonas fluorescens*	Plant protection	[132]
*Serratia marcescens*	Capable of biocontrol against avocado pathogens	[133]
*Aureobasidium pullulans*	Biotechnologically important yeast	[134]
*Penicillium citrinum*	Capable of producing plant growth by promoting metabolites	[135]
*Trichoderma longibrachiatum*	Help optimising culture conditions for agricultural purposes	[73]
**Environmental Contribution**		
*Micrococcus luteus*	Capable of bioremediation of polychlorinated biphenyl (PCB) contaminated environments	[136]
*Pseudomonas putida*	Capable of Xenobiotic degrading	[137]
*Pseudomonas stutzeri*	Capable of denitrification, degradation of aromatic compounds and nitrogen fixation	[138]
*Aureobasidium melanogenum*	Promising biomaterial and can be used for packing food and drugs	[139]
*Rhizopus oryzae*	Capable of biodiesel production	[140]
*Trichoderma viride*	Capable of enhancement of fungal delignification	[75]

Natural products (plants, animals and microorganisms) are essential, reputable resources that originate from Earth’s bio-diverse flora and fauna. These natural products are encoded to be bioactive and have been used as medicines for ages. Today, they continue to be a reservoir of potential resources [141]. Recently, the global threat of anti-microbial resistance has increased the need for urgent therapeutic discoveries and the improvement of existing antimicrobial practices [142]. Numerous medical conditions are the focus of these efforts; however, one of the medical areas in which microorganisms have contributed tremendously in the last few years is cancer research [143]. By loading anti-cancer drugs into nanoparticles, more favourable pharmacokinetics and adjustable biodistribution of nanoparticles can increase the efficacy of the drug [144]. It is noteworthy that the current study detected four microorganisms that have the capability of producing silver nanoparticles. Silver nanoparticles are an arch product from the field of nanotechnology and have gained boundless interest because of their unique properties such as chemical stability, good conductivity, catalytic properties and, most importantly, antibacterial, anti-viral and antifungal activities [145].

In order to make the environment healthier for human beings, contaminated water bodies and land need to be rehabilitated to make them free from toxic waste, heavy metals and trace elements. With the escalated growth of various industries, there has been a considerable increase in the discharge of industrial waste into the air, soil and water, and this has led to the accumulation of heavy metals and toxic waste in these environments, especially in urban areas. The use of microorganisms (*Micrococcus luteus* for example) for remediation technologies and bioremediation to rehabilitate and re-establish the natural condition of the environment is an emerging science [146, 147]. Other ways of environmental rehabilitation using microorganisms, such as fungal delignification (*Trichoderma viride*) [148] and biodiesel production (*Rhizopus oryzae*) [149] have also been investigated during the last few years.

The 26 different useful species that were identified in the selected facility could all be extremely beneficial in various fields of technology; however, not one of these microorganisms was likely to have a direct impact on the product or the food handlers in the facility. Therefore, because there are still no standards or an implementation plan available [150], it is important to create awareness of what needs to be monitored in each industrial environment. Although innocuous and useful bioaerosols do not negatively influence human health, it is critical to mention that the presence of innocuous and useful bioaerosols still serves as an indicator that an ideal environment exists for possible harmful bioaerosols to emerge. In addition, any type of bioaerosol that occurs in excess will have a negative influence on the food product and this should also be considered [20, 109, 150–153].

#### Potentially harmful bioaerosols

Various bioaerosols can have infectious, allergenic or toxic effects on living organisms and may impact human and animal health and agricultural outcomes on a local, regional or global scale. Many plant, animal and human pathogens are dispersed through the air [3, 154], and thus the occupational health of workers is easily affected. Various major infectious diseases in humans such as foot-and-mouth disease, tuberculosis, Legionnaire’s disease, influenza and measles can be spread by airborne bacteria or viruses [4, 77]. Moreover, the inhalation of pathogenic, viable airborne fungi such as *Aspergillus*, *Cryptococcus* and *Pneumocystis* spp. can cause invasive lung infections associated with mortality rates of up to 95% in infected populations [77, 155–157].

Food safety is a complex issue that has an impact on multiple segments of society. Usually a food is considered too adulterated if it contains a poisonous or otherwise harmful substance that is not an inherent natural constituent of the food itself; if it poses a reasonable possibility of injury to health or is presented in a substance that is an inherent natural constituent of the food itself; if it is not the result of environmental, agricultural, industrial, or other contamination; and if is present in a quantity that ordinarily renders the food injurious to health [65].

Harmful microorganisms can: (i) be pathogenic/infectious; (ii) multidrug resistant; (iii) cause food poisoning; (iv) cause food spoilage; (v) cause negative occupational health effects. It was likely that allergenic and/or toxic agents forming bioaerosols and causing occupational diseases of the respiratory tract and skin would be present due to the layout (no airflow, production lines in close proximity to one another) and the type of product the facility produced [56]. Table 4 depicts the four types of harmful bioaerosols that were detected during the two sampling seasons.

**Table 4:** Harmful bioaerosols detected and classified alphabetically according to their pathogenicity and infection potential, multidrug resistance, food poisoning and food spoilage potential.

Genus and Specie	Reference	Genus and Specie	Reference	Genus and Specie	Reference
**Pathogenicity/Infection Potential**					
*Acinetobacter woffii* (MW148768)	[158]	*Pantoea agglomerans* (MW148775)	[57]	*Staphylococcus kloosii* (MW148786)	[159]
*Acinetobacter schindleri* (MW148769)	[160]	*Pseudomonas oryzihabitans* (MW148778)	[161]	*Staphylococcus pasteuri* (MW148787)	[162]
*Aerococcus urinaeequi* (MW148770)	[53]	*Pseudomonas stutzeri* (MW148779)	[138]	*Staphylococcus petrasii (MW148788)*	[163]
*Aerococcus viridans* (MW148771)	[164]	*Serratia marcescens* (MW148780)	[165]	*Staphylococcus saprophyticus* (MW148789)	[166]
*Bacillus licheniformis* (MW148772)	[167]	*Staphylococcus cohnii* (MW148782)	[168]	*Staphylococcus simulans* (MW148790)	[169]
*Brevibacterium casei* (MW148773)	[170]	*Staphylococcus epidermidis* (MW148783)	[171]	*Staphylococcus succinus* (MW148791)	[172]
*Enterobacter hormaechei* (MW148794)	[173]	*Staphylococcus haemolyticus* (MW148784)	[174]	*Staphylococcus succinus subsp*. *casei* (MW148792)	[172]
*Paenibacillus glucanolyticus* (MW148774)	[175]	*Staphylococcus hominis* subsp *novobiosepticus* (MW148785)	[145]	*Staphylococcus warneri* (MW148793)	[176]
*Alternaria* spp. (MW148486)	[64]	*Cryptococcus albidus* (MW165043)	[72]	*Rhodotorula mucilaginosa* (MW165046)	[76]
*Aureobasidium pullulans* (MW165040)	[62]	*Cryptococcus uzbekistanensis* (MW165044)	[177]	*Wickerhamomyces anomalus* (MW165047)	[68]
*Candida intermedia* (MW165041)	[178]	*Rhizopus oryzae* (MW148489)	[69, 179]		
*Candida parapsilosis* (MW165042)	[66]				
**Multidrug Resistance**					
*Staphylococcus arlettae* (MW148781)	[180]	*Staphylococcus epidermidis* (MW148783)	[171]	*Staphylococcus hominis* subsp *novobiosepticus* (MW148785)	[145]
*Staphylococcus cohnii* (MW148782)	[168]	*Staphylococcus haemolyticus* (MW148784)	[174]	*Staphylococcus succinus* (MW148791)	[172]
**Food Poisoning**					
*Bacillus licheniformis* (MW148772)	[167]				
*Penicillium commune* (MW148487)	[168]	*Penicillium crustosum* (MW148488)	[181]		
**Food Spoilage**					
*Pseudomonas fluorescens* (MW148776)	[182]	*Pseudomonas fragi* (MW148777)	[183]		
*Meyerozyma guilliermondii* (MW165045)	[67]	*Penicillium commune* (MW148487)	[168]	*Penicillium crustosum* (MW148488)	[181]

Genbank accession number for each isolate indicated in brackets.

**Fig 4:**
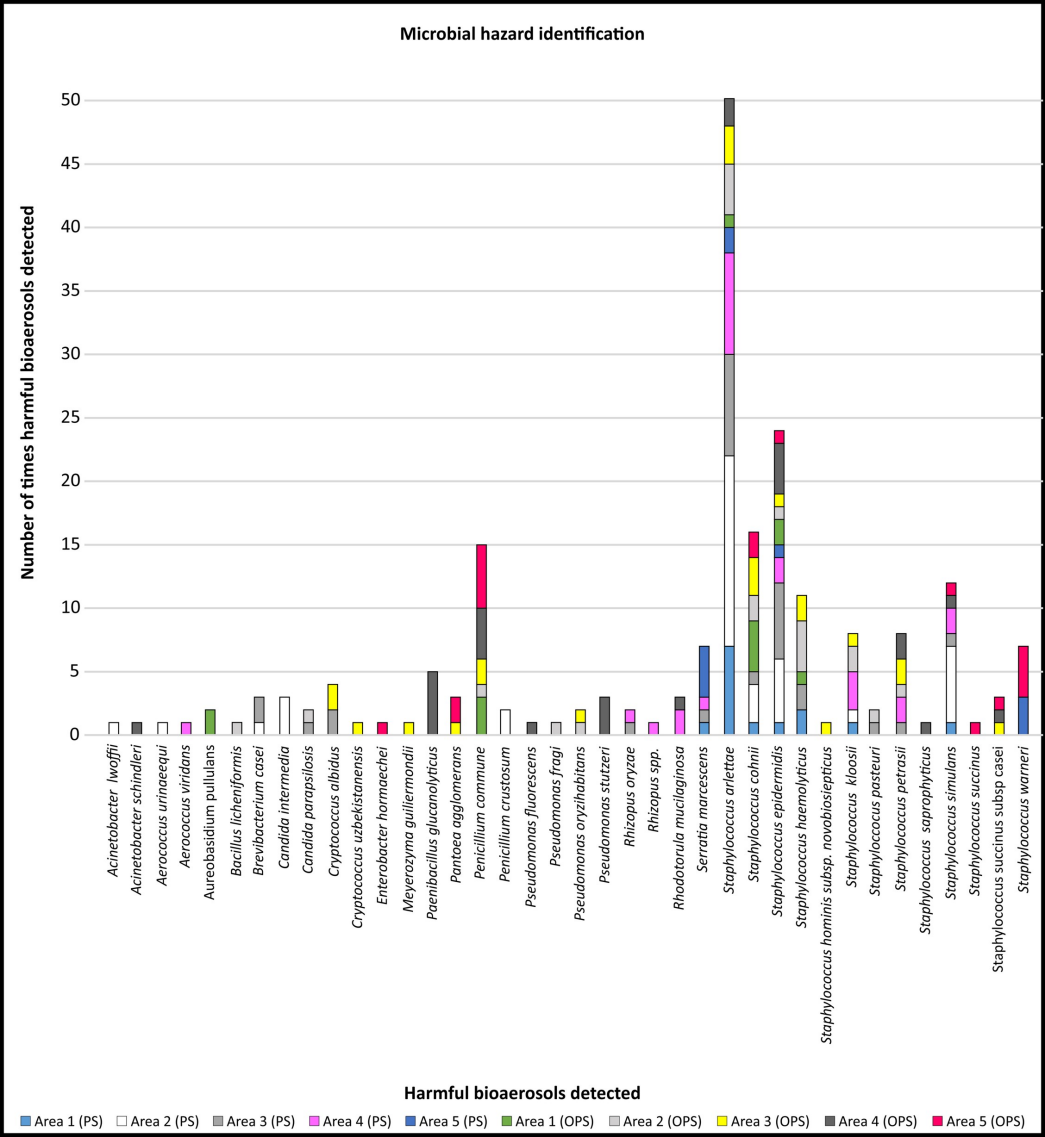
Microbial hazard identification. Number of identified harmful bioaerosols detected during the two sampling seasons in the designated areas: entrance to the production area (Area 1), preparation and mixing of materials (Area 2), between the production lines (Area 3), dispersion of bottles (Area 4) and filling of the final product (Area 5). The two sampling phases are indicated as peak season (PS) (onset of summer) and off-peak season (OPS) (onset of autumn).

*Staphylococcus* spp. are indicators of the severity of air pollution and their presence may indicate the further presence of pathogenic bacteria [184–186]. In the current study, five *Staphylococcus* spp. (*cohnii*, *epidermidis*, *haemolyticus*, *hominis* subsp *novobiosepticus* and *succinus*) were detected on more than ten occasions in different areas in peak and off-peak air samples (Fig 4). *Staphylococcus cohnii*, *epidermidis*, *haemolyticus*, *hominis subsp novobiosepticus* and *succinus* are coagulase-negative staphylococci that may be responsible for bloodstream infections in immuno-suppressed patients [145, 168, 171, 172, 174]. Even though these species can only affect immuno-suppressed individuals, their multidrug resistance capacity against available antimicrobial agents is considered a problem and is the reason why these species are of clinical significance [187].

Although *Staphylococcus* spp. are opportunistic pathogens and rarely cause human infections, their ability to form biofilms on different equipment surfaces had the potential to negatively influence the hygiene of workers in this specific production facility. Despite the low pH as well as the water activity and high sugar content that are characteristics of fruit juice, various *Staphylococcus* spp. have been detected in fruit juice [187–189]. Therefore, according to the standard operating procedures of this specific industry, *Staphylococcus* spp. should not be present in the production environment. Even with regards to infectious diseases only, no clear correlation was found between concentrations of culturable microorganisms in the air and infection. One reason for this could be that infection should be correlated with the dose rather than the concentration. Unfortunately, dose-response relationships still have not been established for most biological agents [47].

*Bacillus licheniformis* is known as one of the most prevalent spore-forming bacterial species which present a worldwide problem to food industries such as fruit juice and dairy manufacturing facilities because of their relevance to food spoilage and quality issues This thermophilic spore-forming bacteria are able to grow during the manufacturing of powder type of products in different sections of manufacturing and will continuum grow throughout processing [190].

Yeast and mould have been used for centuries in the production of diverse foods and beverages. They have also been shown to be involved in the spoilage of an extensive range of foods. Yeasts, predominantly *Saccharomyces* and *Zygosaccharomyces* spp., are able to grow at low pH values in foods with a high sugar content and at refrigeration temperature, making them potential spoilers of refrigerated or concentrated fruit juices [31, 85, 191]. However, none of these yeasts were isolated during the two sampling seasons. This may indicate that they were possibly present but were not isolated; were not present in the environmental air of the facility; or were not present in the environment of this facility at all.

Fungal spoilage encompasses the decay of foods, including the development of off-flavours, acidification, discolouration, and disintegration. Moulds that are typically isolated from fruit juice belong mainly to the *Penicillium* genus and have been identified in several earlier studies [77, 192]. In the current study, *Penicillium commune* was detected on more than ten occasions in peak and off-peak samples collected in different areas (Fig 4). Fungal spoilage can endanger the health of humans by exposing consumers to toxic secondary metabolites such as mycotoxins [5]. The mycotoxin (Cyclopiazonic acid) producing ability displayed by this isolate is a noteworthy fact as it causes poisoning in humans. It is widely known that there is an active metabolism and dissemination of hyaline fungal hyphae inside substrates before the formation of visible colonies on the surface of food. During this period of visible fungus colony formation, there is a potential risk of consumer exposure to mycotoxins [193].

Microorganisms with pathogenicity/infectious capacity, multidrug resistance and food poisoning/spoilage abilities can be found in the air and they also form part of certain environments as bioaerosols. Although the importance of bioaerosols and their impact on human health have been recognised, it is still difficult to accurately describe their role in the initiation or worsening of diverse symptoms and diseases. Diseases and food spoilage arise from exposure to biological agents through the transmission of infectious agents by direct and/or indirect contact, airborne transmission, and vector-borne transmission [194].

The transmission of pathogens and other bioaerosols among humans has been a topic of research for centuries as humans harbour diverse microbes (including pathogens) in and on their bodies. The presence and activities of humans, particularly in indoor environments, can influence bioaerosol concentrations negatively. This is depicted in Fig 4 where, in Area 5 (filling of final product), more personnel were involved and thus higher and more diverse harmful bioaerosols were observed. The emission of particles by breathing, sneezing, coughing, talking and movement, as well as from resuspension of dust due to human activity, has been the focus of numerous indoor bioaerosol studies [5, 151, 195–205]. In high-risk areas, for instance after the last heat treatment before filling and packaging, the food product (beverages) is susceptible to contamination [12].

Apart from the fact that *Candida* spp. and *Staphylococcus* spp. are responsible for a substantial number of infections independently, there is increasing evidence that they can co-exist in cases of biofilm associated infections. Interestingly, in Area 2 and Area 3 where *Candida* spp. were detected, *Staphylococcus* spp. were also observed (Fig 4). The clinical outcome of these mixed bacterial-fungal interactions is that the resultant infections can correlate with an increased frequency or severity of diseases [206]. Staphylococci constitute the main part of the human skin microbiome, and for this reason their role as pathogens has been underestimated [174].

**Fig 5:**
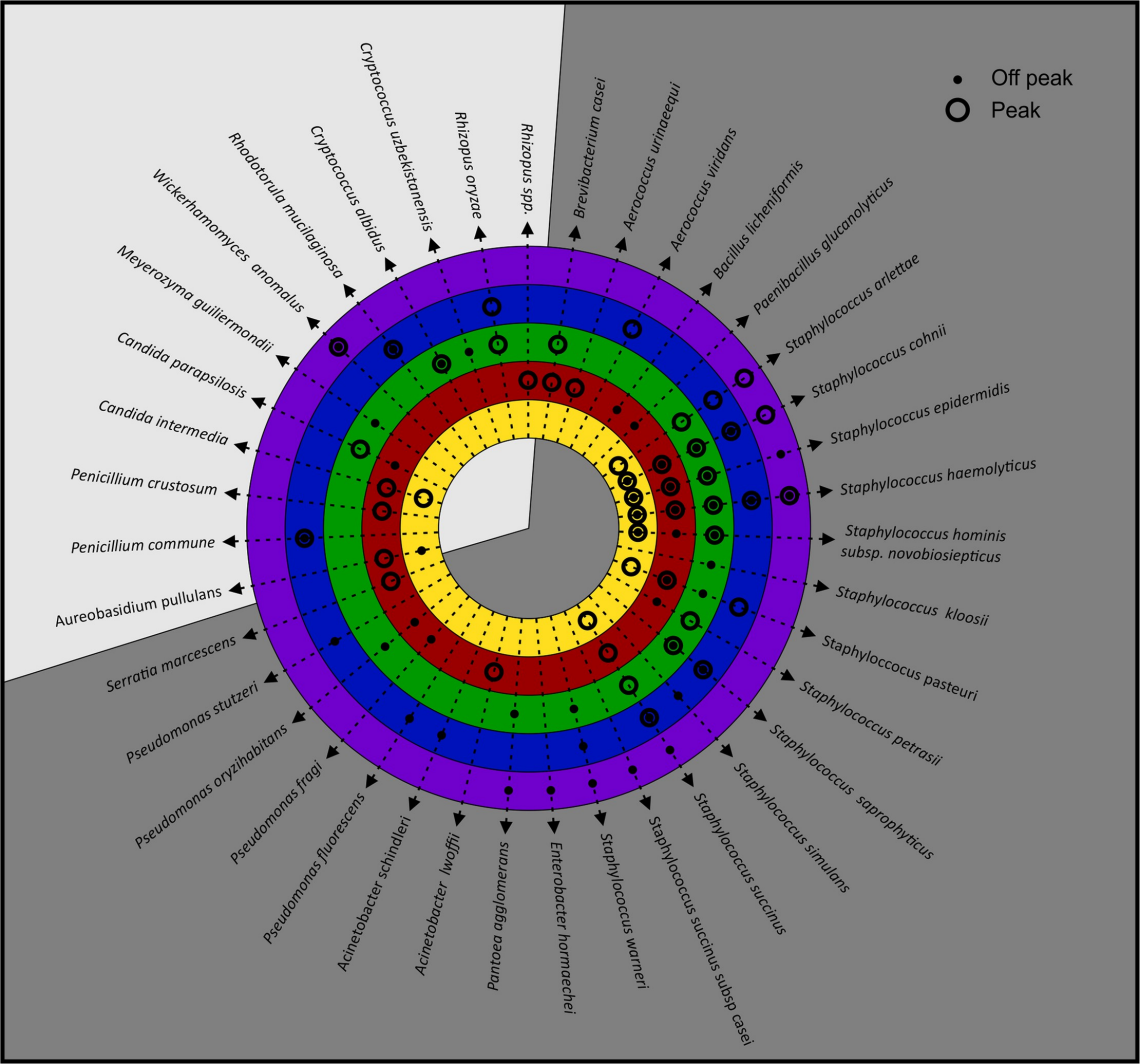
**Harmful bioaerosols detected in samples from the different designated areas: Entrance to the production area (Area 1: Yellow), preparation and mixing of materials (Area 2: Red), between the production lines (Area 3: Green), dispersion of bottles (Area 4: Blue) and filling of the final product (Area 5: Purple).** Sampling occurred during peak season (onset of summer) and off-peak season (onset of autumn) to establish if seasonal variation would impact the accumulation and spread of the harmful bioaerosols. Bacteria are represented by the dark grey region and the yeast and mould are represented by the light grey region.

Climatic conditions have a significant impact on the concentrations and diversity of airborne microorganisms [5, 153, 155]. We considered temperature and airflow to determine if seasonal variation influenced the diversity, distribution and occurrence of harmful bioaerosols, as they were detected in various designated areas in the facility during both seasons (Fig 5). The average indoor air temperature ranged between 18−22°C (±1.1°C) and airflow between 0 to 4.4. m.s^-^1. A clear trend was noted between Area 3 (between the production lines) and Area 4 (dispersion of bottles) with distinguished higher diversity and representability of the same species in both seasons. In Area 5 (filling of final product), where more personnel were involved, higher and more diverse harmful bioaerosols were detected, but the same species were not present during both seasons as the lowest diversity and representability of the same species were observed in Area 1 (entrance to the facility) during both seasons.

Ventilation is one of the most important means to control bioaerosols by supplying outdoor air and remove extra heat, humidity and contaminants from occupied spaces. Deficient ventilation as observed in Area 2 (preparation and mixing of materials) may contribute to massive bioaerosols load and the presence of harmful microorganisms such as *Bacillus licheniformis* and *Penicillium commune* [207].

During both seasons 39 different species were detected; *Staphylococcus* spp. [19] and *Pseudomonas* spp. [6], and to a lesser extent (with two species each) *Aerococcus* spp., *Acinetobacter* spp., *Penicillium* spp., *Candida* spp., *Cryptococcus* spp. and *Rhizopus* spp. were the most prevalently harmful bioaerosols that were identified. Two of these prominent species, namely *Aerococcus* spp. and *Rhizopus* spp., were only detected during the peak season whereas *Acinetobacter* spp., *Penicillium* spp., *Candida* spp. and *Cryptococcus* spp. were detected during both the peak and off-peak seasons.

The second most prominent genera, *Pseudomonas* spp., with a prevalence of the species detected during the off-peak season in Areas 2, 3 and 4, is the most frequently reported genus of the bacteria found after sanitation of food processing surfaces across all types of food production. *Pseudomonas* spp. occur ubiquitously as they are associated with a wide range of niches in food production environments such as fruit juice facilities with respect to nutrients, temperature, surface materials, and stress factors [208]. This genus has established itself on stainless steel coupons placed in the processing environments of fruit juice related industries. *Staphylococcus* spp. is one of the most common Gram-positive genera found in food production environments, and was also the most prominent genera detected during this study in both seasons and were prevalent consistently throughout the facility in all high-risk areas. The biofilm-producing ability of staphylococci may contribute to their persistence in food processing environments, which also occurs in clinical environments [12, 209]. Three *Staphylococcus* spp. (*Staphylococcus cohnii*, *haemolyticus* and *succinus*) were found in all five designated areas.

Bacteria have been reported as the dominant bioaerosol associated microorganisms and they seem to have dominated in most production environments. However, research has shown that in production environments that are more ideal for eukaryotic microorganisms (dry environments and low water activity), yeasts and moulds may be present in significant numbers [209–211]. Four specific eukaryotic microorganisms were detected in this study during both seasons, namely *Cryptococcus albicans*, *Rhodotorula mucilaginosa*, *Wickerhamomyces anomalus*, and *Penicillium commune*.

Even though it is generally accepted that seasonal variations have an influence on the concentration and diversity of microorganisms, any increase in temperature and air exchange rate will cause an increase in airborne bacteria, yeast and mould [5, 20, 152, 153]. We found that the temperature in the studied facility did not fluctuate significantly during the two seasons, and it is thus not unreasonable to assume that external seasonal variation in this case did not influence the microbial concentration or diversity in the different sampling areas. The only variation observed was more personnel present during the off-peak season in all the areas, which might explain the additional species observed during this season. Moreover, the airborne microbial levels increased significantly in the occupied areas compared to the unoccupied areas. This finding supports the argument that humans are mainly the source of bacteria and fungi in settled dust samples [151].

When comparing the densities of the harmful bioaerosols that were detected, only a small group of the species (Table 4) had the ability to influence the products manufactured at the facility. Kim *et al*. [5] argued that although food poisoning and/or spoilage microorganisms are present in the air, it is not a certainty that they will negatively affect the product or consumer. Other factors that affect their capability to cause spoilage or poisoning such as dose relationship, microbial competition and contact with the host should also be considered. With this in mind, it may explain the fact that even though these food poisoning and/or spoilage microorganisms were present in the air, no incidences of product spoilage were reported during routine monitoring at this facility. A significant number of pathogenic bioaerosols were detected, and these all had the potential to impact the occupational health of the personnel in the facility negatively. This confirms the argument that the measurement of bioaerosols should be performed according to a protocol that is representative of exposure patterns and duration that relates to the dose [5, 184]. Therefore, estimating the dose of culturable bacteria that affect people who inhale it in a factory seems to be important for future exposure analyses.

Despite tremendous scientific progress globally, the body of knowledge about biologically originated indoor air pollution seems to remain relatively narrow and insufficient [11]. The reasons for this limited scope could be attributed to: (i) a lack of modern sampling instrumentation (that is industry-bioaerosol specific); (ii) common use of old methods to evaluate the microbiological quality of air; (iii) relatively high costs of instrumental analyses for bacterial and fungal toxins and their markers; (iv) lack of common approved criteria for assessing exposure to biological factors; and (v) a very low number of institutions/organisations interested in (or obligated to perform) comprehensive environmental monitoring of bioaerosols [11].

It has been argued that, although the complexity and importance of the subject of indoor bioaerosol dynamics have been underscored by various studies, our understanding of this phenomenon is not yet mature. One might therefore anticipate fundamental paradigm shifts as knowledge grows and the ability to ask and answer incisive questions improves. Therefore, because the gap between what we know and what we would like to know is extensive, our current knowledge is insubstantial, and we need to realise that we will probably never be able to measure everything. Nevertheless, we need to accurately measure what can reasonably be expected within scientifically determined parameters.

In light of the above arguments, the diversity and complexity of fruit juice facilities will continue to pose great challenges for studies on indoor bioaerosol dynamics. This is because mere basic identification and simply analysing bioaerosol concentrations in the air can lead to misclassification errors of aerosol sources, and misidentification can also lead to misattribution. In this context, the findings of the current study may serve as a reference for future assessments and they may contribute to: (i) policy reviews for product and occupational health; (ii) research efforts in the field to be more outcomes specific; (iii) the implementation of preventative occupational health programs; (iv) the formulation of recommendations aimed at providing healthier production and working environments; and (v) the setting of a clear standard with scientifically established limits in order for facilities to operate within a safe range concerning bioaerosols, the safety of employees, and product quality and safety.

## Conclusion

Bacteria, yeast and mould are the main groups of microorganisms found in bioaerosols. The actual identity, diversity and abundance of different types of bioaerosol particles, as well as their temporal and spatial variability in the fruit juice industry, have not been well characterised. Overall, the role of bioaerosols in the atmosphere and their interaction with other ecosystems are not well described and understood.

The analyses that were conducted isolated 239 bacteria, 41 yeasts and 43 moulds from the air in the production environment of a fruit juice manufacturing facility. The culturable fraction of the bioaerosols identified were categorised into three main groups, namely 27 innocuous, 26 useful and 39 harmful bioaerosols. In the innocuous bioaerosol group, two genera were dominant, namely the *Bacillus* and *Staphylococcus*, and only four innocuous yeasts and moulds were detected. Although innocuous and useful bioaerosols do not negatively influence human health, it is critical to mention that the presence of innocuous and useful bioaerosols serves as an indicator that an ideal environment exists for possible harmful bioaerosols to emerge. In addition, any type of bioaerosol that is in excess could have a negative influence on the food product and should be dealt with.

This study demonstrated that all types of culturable airborne microorganisms occur ubiquitously and are naturally part of the air environment in fruit juice manufacturing facility. It is therefore important that food processing facilities ensure that measures are taken to reduce bioaerosols that may cause product contamination or even occupational health issues. However, there is clearly a need to be more industry- and outcome-specific before monitoring the prevalence of bioaerosols in a specific industry. Culture-dependent methods remain important if information regarding the viability and metabolic activity of these organisms is to be obtained. It is also important that the role that different microbes play in distinctive processes is ascertained and that a clear bioaerosol standard with scientifically established limits be disseminated so that facilities may operate within a safe range.
